# Supplementary figures and images for: Simplified Cell Magnetic Isolation Assisted SC2 Chip to Realize “Sample in and Chemotaxis Out”: Validated by Healthy and T2DM Patients’ Neutrophils
Source: Micromachines (Basel). 2022 Oct 25;13(11):1820. doi: 10.3390/mi13111820 (PMC9692824; doi:10.3390/mi13111820)

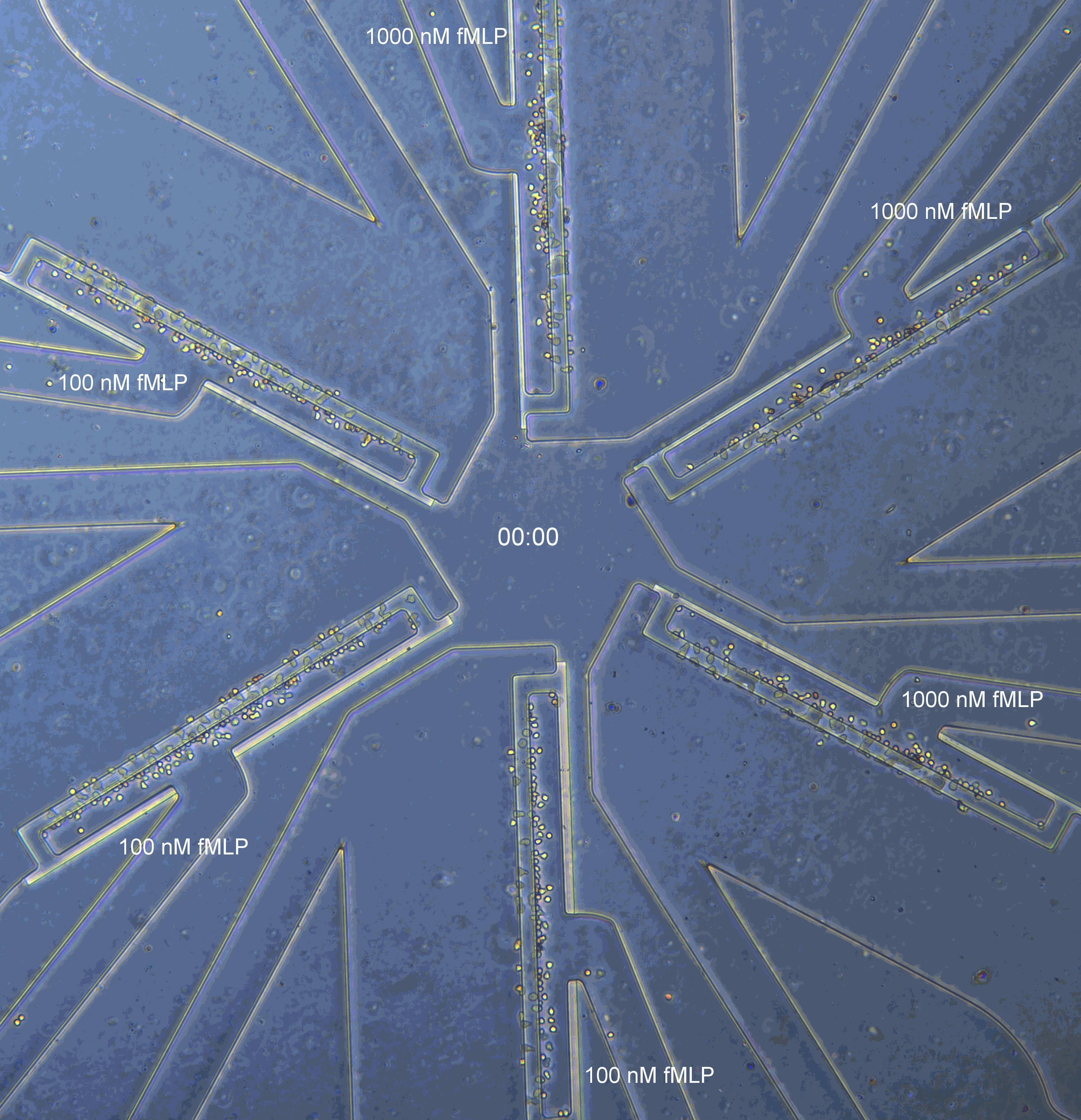

Supplement: Supplementary file 1 [file micromachines-13-01820-s001.zip › micromachines-1981591-supplementary.gif]
